# Overview of major salivary gland cancer surgery in Ontario (2003–2010)

**DOI:** 10.1186/s40463-014-0050-6

**Published:** 2014-12-10

**Authors:** Antoine Eskander, Jonathan Irish, Jeremy Freeman, Patrick Gullane, Ralph Gilbert, Patti A Groome, Stephen F Hall, David R Urbach, David P Goldstein

**Affiliations:** Department of Otolaryngology – Head and Neck Surgery, University of Toronto, Toronto, ON Canada; Department of Otolaryngology – Head and Neck Surgery, Department of Surgical Oncology, Princess Margaret Cancer Centre, Toronto, ON Canada; Department of Otolaryngology – Head and Neck Surgery, Mount Sinai Hospital, Toronto, Ontario Canada; Cancer Care and Epidemiology Division, Queen’s Cancer Research Institute, Queen’s University, Kingston, ON Canada; Department of Otolaryngology – Head and Neck Surgery, Queen’s University, Kingston, ON Canada; Division of General Surgery, Department of General Surgery and Surgical Oncology, University of Toronto, Toronto, ON Canada; Department of Otolaryngology – Head & Neck Surgery, University Health Network, Princess Margaret Hospital, 610 University Ave., Rm 3-952, Toronto, M5G 2M9 ON Canada

**Keywords:** Health services research, Health policy, Salivary gland cancer, Head and neck oncology

## Abstract

**Background:**

The primary objective of this study is to describe variations in incidence rates, resection rates, and types of surgical ablations performed on patients diagnosed with major salivary gland cancers in Ontario.

**Methods:**

All major salivary gland cancer cases in Ontario (2003–2010) were identified from the Ontario Cancer Registry (n = 1,241). Variations in incidence rates, resection rates, and type of surgical therapy were compared by sex, age group, neighbourhood income, community population, health region, and physician specialty.

**Results:**

Eight-year incidence rates per 100,000 vary significantly by sex (male: 15.5, female: 9.7), age (18–54 years: 6.7, 75+ years: 53.4), neighborhood income (lowest quintile: 11.8, highest quintile: 13.7), and community size (cities with a population greater than 1.5 million: 10.6, cities with a population of less than 100,000: 14.7). There was a significant correlation between the likelihood to receive a resection and age with the elderly (75+ years) being the least likely to receive resection (69%). Large differences in incidence and resection rates were observed by health region. Otolaryngology-Head & Neck surgeons provide the majority of total/radical resections (95%).

**Conclusions:**

Major salivary gland cancer incidence rates vary by sex, age, neighborhood income, community size, and health region. Resection rates vary by age and health region. These disparities warrant further evaluation. Otolaryngology-Head & Neck Surgeons provide the majority of major salivary gland cancer surgical care.

## Background

Malignant tumours of the salivary glands have a relatively low incidence as compared to other head and neck cancers. In 2007, 419 new cases of salivary gland cancer were identified in Canada, 230 of which occurred in males [1]. In 2009, 109 deaths were attributable to salivary gland cancer in Canada [1]. U.S. data suggest that the incidence of salivary gland cancers appears to have slightly increased over the last three decades while salivary gland cancer-related deaths have not significantly decreased [2-4]. Salivary gland cancers account for more than 0.5% of all malignancies and approximately 7% of all head and neck cancers [5]. They present largely in the parotid and submandibular glands, require complex multidisciplinary care, and have diverse histology.

The mainstay of treatment is surgical resection with post-operative radiotherapy for those with high risk disease. Distribution and variations in surgical care for major salivary gland cancer (parotid and submandibular) in Ontario, Canada's most populated province with a well organized cancer program, has not been studied since 1997 [6]. This information is critical to support population-based, regional planning of Ontario's cancer surgical services, and to provide background information for provincial quality improvement initiatives in the field of head and neck oncology. The objectives of this study were to describe the incidence of major (parotid and submandibular) salivary gland cancers, and demonstrate the variations in surgical care and hospital level determinants.

## Methods

### Data sources

All malignancies of the parotid and submandibular glands were identified in the *Ontario Cancer Registry* (ICD-9 diagnosis codes: 142.0, 142.1). Validation studies have shown that the OCR is effective at ascertaining cancer cases (98% sensitivity) [7] and specifically for head and neck cancer identifies the cancer site accurately in 91% of primary cases [8]. All non-lymphoma histological types were included according to the ICD-O-3 histology codes. Patients with melanoma were excluded, however, none were detected in our cohort prior to applying exclusion criteria. Despite there being no detected melanoma metastases to the parotid or submandibular gland being miscoded as primary tumours, metastases of unknown primary skin cancers are not distinguishable from primary squamous cell carcinoma of the salivary gland in the registry and, since surgery is the mainstay of treatment for both situations, we included both in this study. Incident major salivary gland cancers cases from the OCR were linked to the *Canadian Institute for Health Information* Discharge Abstract Database (CIHI DAD) and CIHI Same Day Surgery (SDS) Database using relevant salivary gland and submandibular gland resection codes providing hospital level information.

To further ascertain resection, we linked incident head and neck cancer cases from the OCR to Ontario Health Insurance Plan (OHIP) data through which the vast majority of physicians are remunerated for their surgical cases. The OHIP database also provides a unique surgeon identifier which can be linked to the ICES Physician Database (IPDB) to provide information about the physicians performing salivary gland cancer resection including specialty, year of graduation, and gender. The study protocol was approved by the Privacy Office at the Institute for Clinical Evaluative Sciences (ICES) and by the Research Ethics Board of Sunnybrook Health Sciences Centre.

### Study subjects

All Ontario men and women 18 years of age or older who were diagnosed with a non-lymphoma salivary gland cancer between January 1, 2003 and December 31, 2010 were identified in the Ontario Cancer Registry (OCR). This cohort will be referred to as the Overall Salivary Gland Cancer Cohort. From this cohort all patients undergoing resection of the primary tumour site within 12 months before or after their diagnosis date were identified. This cohort will be referred to as the Salivary Gland Resection Cohort. A restrictive resection definition was used to increase confidence in the validity of the resection cohort. For inclusion in the final resection cohort, a patient had to meet the following criteria [1]: identification of their cancer in OCR [2]; resection in CIHI-DAD or SDS databases [3]; resection in OHIP database; and [4] resection dates in CIHI and OHIP must have been within 7 days of each other. After having identified patients with a resection in CIHI-DAD or SDS and then linking to resection codes in OHIP, approximately 10% of patients did not have matching codes. We also performed the reverse and started with OHIP codes and then linked to CIHI-DAD or SDS codes and found that 10% of patients did not have a matching code. As is customary at ICES, we chose to start with CIHI codes as they are considered more reliable. Patients missing key demographic data and those with a prior diagnosis of cancer were excluded.

### Exposures

The following variables were included as important descriptive covariates; sex, age at time of diagnosis, socioeconomic status based on postal code income census data, and community size (>1,500,000; 100,000-1,499,999; and <100,000). Ontario's 14 Local Health Integration Networks (LHINs) which are smaller geographic regions that integrate and distribute health care resources locally, were also used to assess variations in incidence and surgical resection rates.

Ontario has a very highly regionalized head and neck oncology program with 9 hospitals in 6 cities providing the majority of the care, including radiation oncology therapy. These hospitals are well known to the study authors and were designated as head and neck cancer centres. This designation of hospital (head and neck cancer centre or not) was used as a study covariate. Lastly, physicians performing salivary gland cancer surgery were identified by their specialty type, namely, general surgery, plastic surgery, or otolaryngology - head & neck surgery. The specialty designation was determined using the IPDB and OHIP databases. Variations in the use of neck dissection and radiotherapy by LHIN of residence at the time of diagnosis and LHIN of treatment were also examined.

### Outcomes

Incidence rates are presented as 8-year age-standardized incidence rates per 100,000 population. All incidence rates were standardized to the 1991 population of Canada as of July 1, 1991, a year commonly used for analyses of Canadian health data, using the direct method of standardization. Variations in resection rates were assessed for each of the covariates previously described. Finally, the CIHI coding (CCI - Canadian Classification for Health Interventions) for partial resection as opposed to total or radical resection was used to assess differences amongst surgical specialty treating salivary gland malignancies and hospitals in which the procedures were performed. OHIP codes have been found to be inaccurate at determining extent of resection. For this reason we used CIHI codes (CCI - Canadian Classification for Health Interventions) which have been validated for many procedures and have been demonstrated to be far more accurate [9]. The CIHI codes (CCI - Canadian Classification for Health Interventions) used to designate a parotidectomy as radical involve closure of the defect with some form of a reconstructive technique and include both nerve sparing and non-sparing techniques. The remaining CIHI codes for parotidectomy describe a total resection versus a partial resection. We found that extent of parotidectomy from OHIP and CIHI codes were well correlated, that is, more than 90% of OHIP-coded superficial parotidectomies were coded as partial resections whereas OHIP-coded total procedures were coded as either total or radical parotidectomies in CIHI. Because CIHI resection codes (CCI - Canadian Classification for Health Interventions) have been shown to be more accurate, CIHI codes were used. We separated resections as either partial/subtotal or total/radical based on the description provided by CIHI. This applied to both the parotid and submandibular sites and is a measure of extent of resection as coded by CIHI coders. This was used assess whether extent of resection differed by physician specialty and hospital type.

### Statistical analysis

Descriptive analyses are presented by age, gender, neighborhood income quintile, community size, hospital type, surgeon specialty and LHIN. Differences in proportions were tested using the chi square test. For ordinal variables, the Mantel-Haenszel Chi Square was used to assess for trend. Statistical significance was defined by a two-sided p-value of 0.05. All analyses were performed using SAS version 9.2 (SAS Institute, Cary, North Carolina).

## Results

**Figure 1:**
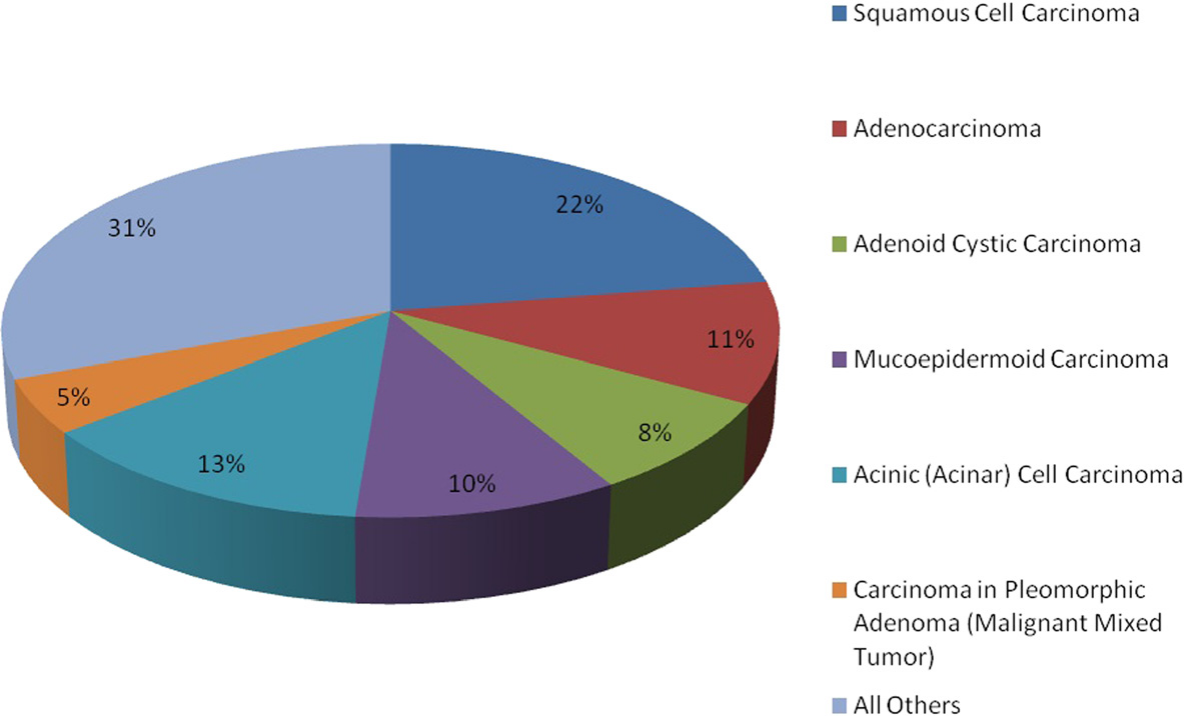
**Salivary gland cancer histology 2003–2010.**

The distribution of histological diagnoses is presented in Figure 1. There were no metastatic melanoma skin cancers incorrectly coded as primary parotid or submandibular gland malignancies. However, 22% of our cohort had squamous cell carcinoma diagnosis.

### Salivary gland incidence

Major salivary gland cancer incidence and surgical resection rates by demographic variables are presented in Table 1. Major salivary gland cancers were more common in males (15.5 per 100,000) and in the older age categories, particularly in those above the age of 70 (30.9 per 100,000 for 70–74 years and 53.4 per 100,000 for those 75 years or over). There were higher incidence rates in higher neighborhood income groups (highest quintile 13.7 per 100,000). There were also higher incidence rates in larger communities (14.7 per 100,000).

**Table 1:** **Salivary gland incidence and surgical intervention by population demographic variables**

	**Incident salivary gland cancer cases**	**8-year age-standardized incidence rate per 100,000**	**Had surgical procedure age-standardized % total**	**Partial or subtotal resection**	**Total or radical resection**
	**n (%)**			**% Total**	**% Total**
**Sex**					
Female	507 (40.9)	9.7	83.6	43.6	40.0
Male	733 (59.1)	15.5	79.9	35.7	44.2
		P < 0.0001	P = 0.10	P = 0.005	P = 0.15
**Age Group**					
18-54 years	409 (33.0)	6.7	91.4	50.1	41.3
55-64 years	246 (19.8)	18.2	82.9	41.5	41.5
65-69 years	92 (7.4)	19.9	83.7	41.3	42.4
70-74 years	122 (9.8)	30.9	81.1	32.8	48.4
75+ years	371 (29.9)	53.4	69.0	26.4	42.6
		P < 0.0001	P < 0.0001	P < 0.0001	P = 0.78
**Neighborhood income**					
1 – Lowest	240 (19.4)	11.8	74.6	34.6	40.0
2	202 (16.3)	9.5	83.7	41.6	42.1
3	260 (21.0)	12.9	82.7	40.0	42.7
4	259 (20.9)	12.6	83.0	39.8	43.2
5 – Highest	279 (22.5)	13.7	83.2	39.1	44.1
		P < 0.0001	P = 0.08	P = 0.67	P = 0.78
**Community population**					
≥ 1,500,000	428 (34.5)	10.6	82.0	38.1	43.9
100,000-1,499,999	474 (38.2)	12.1	82.7	41.4	41.4
< 100,000	338 (27.3)	14.7	79.0	36.7	42.3
		P < 0.0001	P = 0.44	P = 0.41	p = 0.77

### Surgical resection rates and type of resection

Overall, 82% of patients received a surgical resection for their major salivary gland cancer. Resection rates did not vary significantly by sex, however, women were statistically more likely to receive a partial resection as opposed to a radical resection. Increasing age was associated with a statistically significant lower likelihood of receiving a surgery. Radical resection rates did not differ by age category, however, elderly patients (i.e. patients over 75 years of age) were less likely to receive a partial or subtotal resection compared to younger patients. Neighborhood income and community size did not predict likelihood of receiving a surgical procedure or extent of surgery.

### LHIN variations

**Figure 2:**
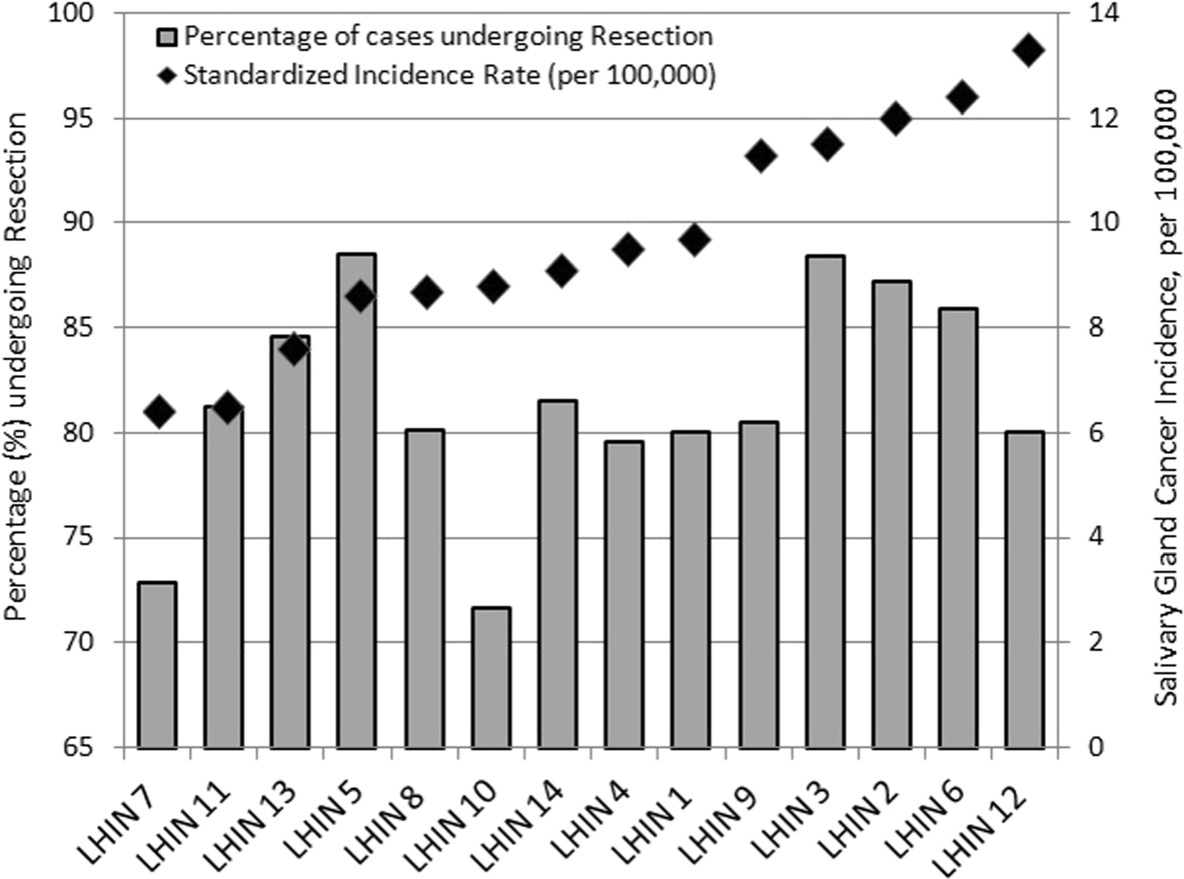
**Salivary gland cancer 8-year incidence and percentage of cases receiving resection within 1 year of diagnosis by Local Health Integration Network (LHIN) of residence at diagnosis.**

There were significant variations (p < 0.001) in major salivary gland cancer incidence rates between Ontario's 14 LHINs as demonstrated in Figure 2. LHIN 7 had an 8-year standardized incidence rate of 6.4 per 100,000 while LHIN 12 had a rate of 13.3 per 100,000. There was also variability between LHINs of residence at time of treatment in terms of surgical resection rates with patients in LHIN 10 having the lowest resection rate (71.7%) and LHIN 8 having the highest (88.5%). This variation was less pronounced than the variation in incidence rates. Surgical resection rates and incidence rates were not correlated. Some LHINs had relatively lower incidence rates with very high resection rates, such as LHIN 5, while some had high incidence rates with relatively low resection rates, such as LHIN 12.

### Surgeon specialty

**Figure 3:**
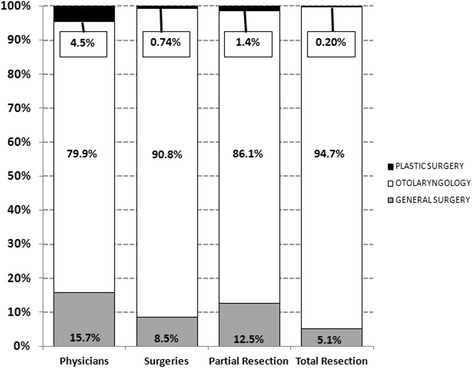
**Percentage of physicians, surgeries, patients, and procedures by specialty.**

Plastic surgeons, general surgeons, and otolaryngologist-head & neck surgeons constituted 4.5%, 15.7%, and 79.9% of surgeons/physicians performing major salivary gland cancer surgery in the province respectively. Plastic surgeons and general surgeons performed a relatively smaller proportion of surgeries 0.74% and 8.5% respectively, as compared to otolaryngologist-head & neck surgeons (90.8%; Figure 3) who operated on the vast majority of patients. Otolaryngologist-head & neck surgeons performed more (94.7%) total or radical resections as compared to plastic surgeons (0.20%) and general surgeons (5.1%).

### Hospital type

The nine designated head and neck cancer centres in Ontario represented only 14.5% of the hospitals performing major salivary gland cancer surgery in the province. There were an additional 53(85.5%) hospitals performing salivary gland cancer surgery. The head and neck cancer centres provided care to a disproportionate number of cases, operating on 57.4% of our patient cohort. Furthermore, these centres were far more likely to perform total or radical resections (60.3% of their cases) as compared to other centres (only 41.2% of their cases) (Table 2).

**Table 2:** **Salivary gland cancer pattern of surgical care by hospital type**

		**Definitive procedure by hospital type**	
**Hospital type**	**Total number**	**Partial or subtotal resection**	**Total or radical resection**
	**N (% total)**	**n (% resections)**	**n (% resections)**
Head & neck cancer centre	580 (57.4)	39.7	60.3
Other	430 (42.6)	58.8	41.2
P-value		<0.0001	
Total	1010 (100)	47.8	52.2

### Neck dissection and post-operative radiotherapy by LHIN of residence and treatment

There were some variations noted in the use of neck dissection and radiotherapy by LHIN of residence and treatment (Tables 3 and 4). On average, 49.2% of patients that received a resection also received a neck dissection. Patients residing in LHIN 12 at the time of their diagnosis received the most neck dissections (62.5%) while those in LHIN 10 had the lowest neck dissection rates (37.2%); however, differences in neck dissection by LHIN of residence were not statistically significant (p-value 0.21). On average, 59.3% of patients that received a resection also received radiotherapy. Patients residing in LHIN 14 at the time of their diagnosis were most likely to receive radiotherapy (77.2%) while those in LHIN 10 were least likely to have received radiotherapy (46.5%); however, differences in radiotherapy use by LHIN of residence were not statistically significant (p-value 0.16). More dramatic variations in neck dissection (p-value <0.001) and radiotherapy rates (p-value <0.001) were noted by LHIN of treatment (Table 4).

**Table 3:** **Variations in the use of neck dissection and post-operative radiotherapy by LHIN of residence**

**LHIN of residence**	**Sample size**	**% who received neck dissection**	**% who received radiotherapy**
Resection Cohort	1010	49.2	59.3
LHIN 1	60	50.0	56.7
LHIN 2	109	47.7	55.0
LHIN 3	61	47.5	55.7
LHIN 4	113	46.9	57.5
LHIN 5	46	43.5	60.9
LHIN 6	79	53.2	46.8
LHIN 7	67	56.7	62.7
LHIN 8	101	58.4	62.4
LHIN 9	136	44.9	61.0
LHIN 10	43	37.2	46.5
LHIN 11	78	39.7	65.4
LHIN 12	40	62.5	67.5
LHIN 13	55	56.4	69.1
LHIN 14	22	45.5	77.3

**Table 4:** **Variations in the use of neck dissection and post-operative radiotherapy by LHIN of treatment**

**LHIN of treatment**	**Sample size**	**% who received neck dissection**	**% who received radiotherapy**
Resection cohort	1010	49.2	59.3
LHIN 1	22	27.3	40.9
LHIN 2	160	52.5	58.8
LHIN 3	33	21.2	42.4
LHIN 4	125	50.4	55.2
LHIN 5	28	28.6	50.0
LHIN 6	55	43.6	50.9
LHIN 7	281	73.3	69.4
LHIN 8	33	18.2	57.6
LHIN 9	82	28.0	50.0
LHIN 10	27	18.5	40.7
LHIN 11	87	40.2	65.5
LHIN 12	18	27.8	44.4
LHIN 13	45	51.1	68.9
LHIN 14	14	14.3	64.3

## Discussion

Major salivary gland cancer incidence increases with age. The elderly represent nearly 40% of those with major salivary gland cancer in our cohort and are far less likely to receive surgical resection. The mainstay of treatment for major salivary gland cancers remains surgical resection with or without post-operative radiotherapy. The elderly are likely to have more comorbidities, are much more likely to experience acute medical complications (OR 3.7), in-hospital death (OR 3.6), increased hospitalization (by a mean of 2.2 days), and increased hospital-related costs (by a mean of $6,874 US) as demonstrated using a cohort of head and neck cancer patients derived from the Nationwide Inpatient Sample [10]. Also, elderly patients treated for major salivary gland cancer have a poorer prognosis independent of treatment [11]. For these reasons, surgical treatment may be precluded more often in the elderly patients.

Major salivary gland cancer incidence was also associated with increasing neighbourhood income quintile and smaller community size, a finding which has never been previously reported in the medical literature. This is the opposite of trends described for mucosal head and neck malignancies [12,13]. It has been demonstrated that Caucasians are more likely to develop major salivary gland cancer than other racial groups, and this measure of socioeconomic status could potentially be related to race, a variable not available in Canadian health administrative databases [4]. Certain occupations are however associated with salivary gland cancer including rubber products manufacturing, asbestos mining, plumbing and some types of woodworking [14]. There has been no definitive evidence in the literature that major salivary gland cancer or that any cancer for that matter, is related to cell phone use [15,16], however, given the location of these glands, and the higher incidence in higher socioeconomic groups, this may warrant further study.

There are significant variations by geographic region of residence (ie. LHIN) with regards to incidence, resection rates, neck dissection and radiotherapy use. The variation in incidence across geographic region may be due to a variety of etiologies which our current dataset is unable to capture including varying infection rates, environmental exposures, carcinogenic exposures or this may be due to confounding by our inclusion of metastatic skin squamous cell carcinoma. It is worth noting that these variations are striking and significantly greater than for other cancers in our province [9]. The variations in neck dissection and radiotherapy use may be appropriate based on histological type and extent of disease but such large variations merit further study. Pathological staging data is starting to become available and will be the subject of future research which may help answer this question.

Our data suggest that the management of salivary gland cancers in Ontario is far less regionalized than for oral cavity and larynx/hypopharynx cancer. For both of these latter subsites, greater than 90% of cases are performed at head and neck cancer centres as compared to the 57% demonstrated for salivary gland cancers. We have previously reviewed volume-outcome studies in the field of head and neck oncology, and concluded that there lacked evidence for such a relationship in the treatment of major salivary gland cancers [17]. Despite ultrasound-guided fine needle aspiration and trained cytologists, a significant proportion of salivary gland lesions are excised without a pre-operative tissue diagnosis and are thus performed for diagnostic and therapeutic purposes. Ultrasound-guided fine needle aspiration and advanced cytological diagnosticians are not readily available in many regions in the province of Ontario and this may affect surgeons’ ability to pre-operatively plan the extent of resection. However, the data on neck dissection and radiotherapy by region (ie. LHIN) of treatment demonstrates much higher rates of both of these interventions in regions with a head and neck cancer centre. This suggests that advanced and/or aggressive tumours, based on clinical and radiological grounds, are being appropriately referred to high volume centres for resection and subsequent management. Although the preliminary data suggests this, further study is required to confirm whether this is indeed the case as this was not the primary objective of our study. Pathologic and staging data which is becoming increasingly available in our datasets will help elucidate this. Given the rarity of these tumours, their varied histologies and the current lack of regionalization as compared to the management of other head and neck cancer subsites, a policy to further regionalize the treatment of these tumours may be warranted.

These data must be interpreted in the context of the study design. The most important limitation of this study is the possibility of the OCR having misidentified skin squamous cell carcinoma metastatic to the parotid gland as primary parotid gland malignancies. Although this is possible, we do not feel that this changes the importance of our findings, which in the context of a universal health care system have significant implications. Also, this weakness is mitigated by the fact that patients with skin squamous cell carcinomas in close proximity to or metastatic to the parotid may receive a parotidectomy and neck dissection with or without adjuvant treatment, a similar treatment paradigm to most primary parotid malignancies. Nonetheless, the squamous cell carcinoma histology may have significant impacts on our variations by LHIN of residence as some of the regions with the highest incidence rates are largely Caucasian, and have a higher population of outdoor workers (e.g. farmers), important risk factors for sun-induced skin cancers. Another important limitation is our inability to determine resection rates by LHIN of treatment since we have little data on those patients that did not receive surgical resection, limiting our ability to determine a denominator (ie. total number of patients presenting with cancer) for each LHIN of treatment.

Our study has a number of strengths. The OCR has a much higher cancer capture rate than the two most commonly used U.S. databases (SEER and NCDB; 26% and 70% respectively). This makes the OCR more representative of Ontario patients than SEER or NCDB for U.S. populations. This study is the largest series in the literature describing important sociodemographic factors in major salivary gland cancer patients. A number of key variations have been identified, which raises concerns around equitable treatment of patients with salivary gland cancer across our province and these merit further inquiry with the aim of reducing such variations. This type of overview is particularly useful for policy makers and health administrators as resources are organized and is the first step towards improving access and quality of care.

Our findings have important implications for policy makers as it relates to two of Canada Health Act's key tenants; universality and equitability. Surgery remains the mainstay of treatment for major salivary gland cancers. There appears to be a relatively low resection rates in certain regions (LHINs) and in certain populations (the elderly). These findings require further investigation but raise important questions about access to Otolaryngology - Head & Neck surgery expertise in our province. Certain LHINs have lower resection rates and one possible hypothesis is lack of access to major salivary gland cancer surgery expertise. Similarly, further study is required to determine the appropriateness of lower resection rates in the elderly. Although these resection rates may be appropriate, given our aging population and the higher incidence of major salivary gland cancers in the elderly, this will be an ongoing equitability concern.

## Conclusion

Major salivary gland cancer incidence rates vary by sex, age, neighborhood income, community size, and health region. Resection rates vary by age and health region. These disparities warrant further evaluation to determine whether there is access inequity and whether there are quality of care improvements that can be made in this area of care.

## Consent

Written informed consent was not obtained from the patients included in this study because administrative de-identified data was used.

## Abbreviations

OCR: Ontario cancer registry
CIHI: Canadian Institute for Health Information
OHIP: Ontario health insurance plan
ICES: Institute for Clinical Evaluative Sciences
IPDB: ICES Physician Database
